# Supplementary material for: The Relationships between Caregivers’ Concern about Child Weight and Their Non-Responsive Feeding Practices: A Systematic Review and Meta-Analysis
Source: Nutrients. 2022 Jul 14;14(14):2885. doi: 10.3390/nu14142885 (PMC9323971; doi:10.3390/nu14142885)
Supplement: Supplementary file 1 [file nutrients-14-02885-s001.zip › Supplementary Table S1.pdf]

Supplementary Table S1. Quality appraisal by the JBI Critical Appraisal Checklist for Analytical Cross-Sectional Studies

	1. Were the criteria for inclusion in the sample clearly defined?	2. Were the study subjects and the setting described in detail?	3. Was the exposure measured in a valid and reliable way?	4. Were objective, standard criteria used for measurement of the condition?	5. Were confounding factors identified?	6. Were strategies to deal with confounding factors stated?	7. Were the outcomes measured in a valid and reliable way?	8. Was appropriate statistical analysis used?	Overall appraisal
Xiang, 2021 [83]	Y	Y	Y	NA	Y	Y	Y	Y	Include
Branch,2017 [65]	Y	Y	Y	NA	Y	Y	Y	Y	Include
Francis, 2001 [24]	Y	Y	Y	NA	Y	Y	Y	Y	Include
Freitas, 2019 [79]	Y	Y	Y	NA	Y	Y	Y	Y	Include
Webber, 2010 [86]	Y	Y	Y	NA	Y	Y	Y	Y	Include
Gebru, 2021 [29]	Y	Y	Y	NA	Y	Y	Y	Y	Include
de Souza Rezende, 2019 [50]	Y	Y	Y	NA	Y	Y	Y	Y	Include
Mais, 2017 [28]	Y	Y	Y	NA	Y	Y	Y	Y	Include
Cachelin, 2013 [66]	Y	Y	Y	NA	Y	Y	Y	Y	Include
Ek, 2016 [80]	Y	Y	Y	NA	Y	Y	Y	Y	Include
Eli, 2016 [81]	Y	Y	Y	NA	Y	Y	Y	Y	Include
Gregory, 2010 [77]	Y	Y	Y	NA	Y	Y	Y	Y	Include
Wang, 2022 [84]	Y	Y	Y	NA	Y	Y	Y	Y	Include
Haines, 2018 [27]	Y	Y	N	NA	Y	Y	Y	Y	Include
Bouhlal, 2018 [67]	Y	Y	Y	NA	Y	Y	Y	Y	Include
de Lauzon-Guillain, 2009 [74]	Y	Y	Y	NA	Y	Y	Y	Y	Include
Srivastava, 2021 [21]	Y	Y	Y	NA	Y	Y	Y	Y	Include
Brann, 2010 [68]	Y	Y	Y	NA	N	N	Y	Y	Include
Warkentin, 2018 [30]	Y	Y	Y	NA	Y	Y	Y	Y	Include
Tan, 2011 [69]	Y	Y	Y	NA	N	N	Y	Y	Include

	1. Were the criteria for inclusion in the sample clearly defined?	2. Were the study subjects and the setting described in detail?	3. Was the exposure measured in a valid and reliable way?	4. Were objective, standard criteria used for measurement of the condition?	5. Were confounding factors identified?	6. Were strategies to deal with confounding factors stated?	7. Were the outcomes measured in a valid and reliable way?	8. Was appropriate statistical analysis used?	Overall appraisal
Somaraki, 2017 [22]	Y	Y	Y	NA	Y	Y	Y	Y	Include
Salinas Martínez, 2020 [87]	Y	Y	N	NA	Y	Y	Y	Y	Include
Rodgers, 2013 [75]	Y	Y	Y	NA	Y	Y	Y	Y	Include
Loth, 2021 [70]	Y	Y	Y	NA	Y	Y	Y	Y	Include
Mallan, 2014 [76]	Y	Y	Y	NA	Y	Y	Y	Y	Include
Chae, 2018 [88]	Y	Y	N	NA	Y	Y	Y	Y	Include
May, 2007 [71]	Y	Y	Y	NA	Y	Y	Y	Y	Include
Seburg, 2014 [72]	Y	Y	Y	NA	Y	Y	Y	Y	Include
Crouch, 2007 [78]	Y	Y	Y	NA	Y	Y	Y	Y	Include
Ayine, 2020 [73]	Y	Y	Y	NA	N	N	Y	Y	Include
Jani Mehta, 2014 [25]	Y	Y	Y	NA	Y	N	Y	N	Include
Nowicka, 2014 [82]	Y	Y	Y	NA	Y	Y	Y	Y	Include
