# Supplementary material for: The Relationships between Caregivers’ Concern about Child Weight and Their Non-Responsive Feeding Practices: A Systematic Review and Meta-Analysis
Source: Nutrients. 2022 Jul 14;14(14):2885. doi: 10.3390/nu14142885 (PMC9323971; doi:10.3390/nu14142885)
Supplement: Supplementary file 1 [file nutrients-14-02885-s001.zip › Supplementary Table S3.pdf]

Supplementary Table S3. Related measurements and the estimates of the relationships between caregivers' concern about child weight and their non-responsive feeding practices (n = 35)

**First author, year**	**Exposure and measure**	**Outcome and measure**	**Result and Estimates**	**Statistical Analysis and covariates in the model**
Xiang, 2021 [83]	CFQ (one item)<br>Concern about child overweight (Unconcerned V.S. concerned)	C-CFQ<br>1) Restriction<br>2) Food as reward<br>3) Pressure to eat<br>Continuous variable	Underweight children (*n*=222)<br>1) β = -0.244 (95%CI: -0.484, -0.005), *P* < 0.05<br>2) β = -0.154 (95%CI: -0.418, 0.111)<br>3) β = -0.119 (95%CI: -0.337, 0.098)<br>Normal weight children (*n*=911)<br>1) β = 0.123 (95%CI: 0.010, 0.236), *P* < 0.05<br>2) β = 0.002 (95%CI: -0.124, 0.129)<br>3) β = 0.025 (95%CI: -0.084, 0.135)<br>Overweight/obese children (*n*=355)<br>1) β = 0.052 (95%CI: -0.153, 0.258)<br>2) β = -0.141 (95%CI: -0.347, 0.091)<br>3) β = -0.146 (95%CI: -0.350, 0.057)	Multiple linear regression<br><br>Child gender, age, and only child, and parental role, and education.
Branch,2017 [65]	CFQ (one item)<br>Concern about child overweight (Unconcerned V.S. some concerned V.S. high concerned)	CFQ<br>1) Restriction<br>2) Pressure to eat<br>Continuous variable	Mean score (SE)<br>1) 3.1 (0.1) V.S. 3.5 (0.1) V.S. 3.6 (0.1), *P* = 0.004<br>2) 2.7 (0.1) V.S. 2.8 (0.1) V.S. 2.8 (0.2), *P* = 0.96	MANOVA<br><br>Child gender, age, race/ethnicity, BMI z-score, and maternal education and BMI.
Francis, 2001 [24]	CFQ (four items)<br>Concern for child weight (Continuous variable)<br><br>Mothers' concern was indicated by four items from the Concerns about Child Overweight subscale from the CFQ.	CFQ<br>1) Restriction<br>2) Pressure to eat<br>Continuous variable	1) β = 0.18, *P* > 0.05<br>2) β = 0.13, *P* > 0.05	SEM<br><br>Covariates: Maternal weight concern and restraint, daughters' adiposity, family income, maternal education, maternal depression, general parenting style
Freitas, 2019 [79]	CFQ (one item)<br>Concern about child overweight (Unconcerned V.S. concerned)	CFPQ<br>Restriction (for weight control)	OR = 2.61 (95%CI: 1.938, 3.511)<br>*P* < 0.001	Multiple logistic regression<br>Covariates: mother's BMI, child's age and gender
Webber, 2010 [86]	One item (5-point Likert scale)<br>Maternal concern about child overweight (unconcerned V.S. a little concerned V.S. concerned)	CFQ<br>1) Restriction<br>2) Pressure to eat<br>Continuous variable	1） F (2,188) = 9.60, *P* < 0.001<br>2） *P* > 0.05	Trend analysis<br>Covariates: child sex and age, maternal ethnicity and education)

First author, year	Exposure and measure	Outcome and measure	Result and Estimates	Statistical Analysis and covariates in the model
Gebru, 2021 [29]	CFQ (three item)<br>Caregivers' concern about child overweight<br>PFQ (three item)<br>Concern about underweight<br>(Continuous variable)	CFQ<br>1) Restriction<br>2) Pressure to eat<br>Continuous variable	Concern about child overweight<br>1) β = 0.11 (95%CI: 0.04, 0.18), *P* < 0.01<br>2) β = -0.1 (95%CI: -0.17, -0.02), *P* < 0.01<br>Concern about child underweight<br>1) β = 0.01 (95%CI: -0.04, 0.07)<br>2) β = 0.15 (95%CI: 0.08, 0.21), *P* < 0.01	Multiple linear regression<br><br>Covariates: child's age, child's sex, child's BMI z score, child's mental health status, caregiver's depression status, caregiver's perceived feeding responsibility, perceived caregiver weight, concern about child weight, caregivers' education level and socioeconomic status
de Souza Rezende, 2019 [50]	Adapted from CFQ (one item)<br>Maternal concern about child underweight (concern about underweight V.S. concern about non-underweight)	CFPQ<br>Pressure to eat	OR=1.63 (95%CI: 1.21, 2.18), *P* < 0.01	Bivariate analyses<br>Not control for covariates
Mais, 2017 [28]	CFQ (three items)<br>Parental concern about child overweight (concerned V.S. unconcerned)<br>Parental concern about child underweight (concerned V.S. unconcerned)	CFPQ<br>1) Restriction for weight<br>2) Restriction for health<br>3) Pressure to eat<br>4) Use food as a reward	Parental concern about child overweight<br>1) OR = 2.98, *P* < 0.001<br>2) OR = 1.88, *P* < 0.001<br>3) NR<br>4) NR<br>Parental concern about child underweight<br>1) NR<br>2) NR<br>3) OR = 1.49, *P* = 0.014<br>4) NR	Multivariate logistic regression<br><br>Covariates: child sex and maternal education
Cachelin, 2013 [66]	CFQ (three items)<br>Concern about child weight<br>(Continuous variable)	CFQ<br>Control of child eating (Restriction + Pressure + Monitoring)	Hispanic model (*n*=218)<br>Standardized β = 0.30, *P* < 0.05<br>White model (*n*=115)<br>Standardized β = 0.45, *P* < 0.05	SEM<br><br>Annual household income, child age, maternal BMI
Ek, 2016 [80]	CFQ (three items)<br>Concern about child weight<br>(Continuous variable)	CFQ<br>1) Restriction<br>2) Pressure to eat<br>Continuous variable	1) β = 0.58, *P* < 0.05<br>2) β = 0.13, *P* > 0.05	SEM<br>Child age, gender, and body mass index standard deviation score and parental age, gender, body mass index, foreign origin, and education level as well as for

First author, year	Exposure and measure	Outcome and measure	Result and Estimates	Statistical Analysis and covariates in the model
				parental life-style specific Confidence
Derks, 2017 [85]	CFQ (two items)<br>Concern about child overweight weight (Continuous variable)	CFQ<br>Restriction<br>Continuous variable	Model 1: β = 0.35 (95%CI: 0.32, 0.38)<br>Model 2: β = 0.33 (95%CI: 0.30, 0.36)<br>Model 3: β = 0.36 (95%CI: 0.33, 0.38)	SEM<br>Child ethnicity, birth weight, household income, maternal BMI, maternal depressive symptoms
Eli, 2016 [81]	CFQ (three items)<br>Concern about child weight (Continuous variable)<br>Parents are concerned about their child becoming overweight or eating foods with too much fat or sugar	CFQ<br>1) Restriction<br>2) Pressure to eat	1) β = 0.32, $P < 0.05$<br>2) β = 0.01, $P > 0.05$	SEM<br>Child sex, child's age, child's BMI, mother's foreign background, mother's level of education, and mother's BMI
Gregory, 2010 [77]	CFQ (three items)<br>Concern about child overweight<br>PFQ (two items)<br>Concern about child underweight (Continuous variable)	CFQ<br>1) Restriction<br>2) Pressure to eat<br>Continuous variable	Concern about child overweight<br>1) β = 0.18, $P = 0.025$<br>2) NR<br>Concern about child underweight<br>1) NR<br>2) β = 0.39, $P < 0.01$	Hierarchical multiple regression<br><br>Maternal BMI and education
Wang, 2022 [84]	CFQ (one item)<br>Concern about child overweight (Continuous variable)	C-CFQ<br>1) Restriction<br>2) Reward	1) β = 0.115 (95%CI: 0.057, 0.168)<br>2) β = -0.069 (95%CI: -0.131, 0.004)	SEM<br>Child age, maternal age, maternal education level and annual household income
Haines, 2018 [27]	One item<br>Maternal concern about child overweight (unconcerned V.S. concerned)<br>One item<br>Maternal concern about child underweight (unconcerned V.S. concerned)	CFPQ<br>1) Pressure<br>2) Restriction<br>3) Reward<br>4) Emotional regulation	Concern about child overweight<br>1) β = -0.41 (95%CI: -0.91, 0.01)<br>2) β = 0.54 (95%CI: 0.20, 0.86)<br>3) β = -0.22 (95%CI: -0.77, 0.31)<br>4) β = 0.01 (95%CI: -0.35, 0.36)<br>Concern about child underweight<br>1) β = 0.70 (95%CI: 0.30, 1.1)<br>2) β = 0.56 (95%CI: 0.20, 0.91)<br>3) β = 0.49 (95%CI: 0.08, 0.89)<br>4) β = 0.08 (95%CI: -0.14, 0.30)	Multivariable linear regression analyses<br><br>Maternal education, language spoken at home, maternal weight status, treatment condition.
Bouhlal, 2018 [67]	CFQ (three items)<br>Concern about child weight (Continuous variable)	CFQ<br>Restriction<br>Continuous variable	β = 0.25, $P < 0.0001$	SEM<br>Maternal education level and perception of the child's weight

First author, year	Exposure and measure	Outcome and measure	Result and Estimates	Statistical Analysis and covariates in the model
de Lauzon-Guillain, 2009 [74]	CFQ (one item)<br>Concern about child overweight<br>(Continuous variable)	CFPQ<br>1) Restriction for weight<br>2) Restriction for health<br>3) Emotional regulation<br>4) Reward<br>Continuous variable	1) β = 0.4 (SE: 0.1), *P* < 0.01<br>2) β = 0.4 (SE: 0.1), *P* < 0.01<br>3) β = 0.00 (SE: 0.1)<br>4) β = 0.00 (SE: 0.1)	Multivariable linear regression analyses<br><br>Parental sex, country of residence
Srivastava, 2021 [21]	CFQ (three items)<br>Concern about child weight<br>(Continuous variable)	CFQ<br>1) Pressure to eat<br>2) Restriction	1) β = -0.18 (SE: 0.09) (95%CI: -0.45, -0.09)<br>2) β = 0.22 (SE: 0.07) (95%CI: 0.13, 0.39)	Multivariable linear regression analyses<br>Parental age, weight, child age, gender, and household income
Brann, 2010 [68]	CFQ (three items)<br>Concern about child weight<br>(Continuous variable)	CFQ<br>1) Pressure to eat<br>2) Restriction	1) r = 0.53, *P* < 0.01<br>2) r = 0.27, *P* < 0.01	Pearson correlation<br><br>Not control for covariates
Webb, 2019 [26]	CFQ (three items)<br>Concern about child weight<br>(Continuous variable)	CFPQ<br>1) Restriction for weight<br>2) Restriction for health<br>3) Pressure to eat<br>Continuous variable	1) β = -0.03 (SE: 0.07)<br>2) β = 0.03 (SE: 0.16)<br>3) β = -0.01 (SE: 0.11)	Multiple regression analyses<br>T1 parental body dissatisfaction, restriction for child weight/health and pressure to eat.
Warkentin, 2018 [30]	CFQ (three items)<br>Concern about child overweight<br>(concerned V.S. unconcerned)<br>Concern about child underweight<br>(concerned V.S. unconcerned)	CFPQ<br>1) Restriction for weight<br>2) Restriction for health<br>3) Pressure to eat<br>4) Food as a reward	Parental concern about child overweight<br>1) OR = 2.46 (95%CI: 1.64, 3.69)<br>2) NR<br>3) NR<br>4) NR<br>Parental concern about child underweight<br>1) NR<br>2) NR<br>3) OR = 2.30 (95%CI: 1.53, 3.47)<br>4) NR	Multiple logistic regression models<br><br>Child sex, maternal education, BMI, perceived responsibility for child feeding.
Tan, 2011 [69]	CFQ (three items)<br>Concern about child weight<br>(continuous variable)	CFPQ<br>1) Restriction for health<br>2) Restriction for weight	1) r = 0.46, *P* < 0.01<br>2) r = 0.34, *P* < 0.05	Pearson correlation<br><br>Not control for covariates
Somaraki, 2017 [22]	CFQ (three items)<br>Concern about child weight<br>(Continuous variable)	CFQ<br>1) Restriction<br>2) Pressure to eat	1) β = 0.42 (95%CI: 0.35, 0.50)<br>2) β = -0.14 (95%CI: -0.22, -0.06)	Multiple regression models<br><br>Child sex, age, maternal age, education, weight status.

First author, year	Exposure and measure	Outcome and measure	Result and Estimates	Statistical Analysis and covariates in the model
Salinas Martínez, 2020 [87]	Concern about child overweight (concerned V.S. unconcerned)	1) CFQ+CFPQ: Pressure to eat<br>2) PFQ+CFPQ: Regulation<br>3) CFQ+CFPQ: Restriction	1) Adjusted OR=0.7 (95%CI: 0.5, 1.1)<br>2) Adjusted OR=0.8 (95%CI: 0.5, 1.3)<br>3) Adjusted OR=1.5 (95%CI: 1.1, 2.3)	Multivariate logistic regression analysis<br><br>Covariates: child weight concern, sex, birth order and age; mother's overweight/obesity and age
Rodgers, 2013 [75]	CFQ (three items)<br>Concern about child weight (Continuous variable)	CFPQ<br>Restriction	β = 0.25, *P* < 0.001	SEM<br>Maternal BMI, body dissatisfaction and maternal dietary restraint
Loth, 2021 [70]	CFQ (two items)<br>Concern about child (over)weight (Not concerned V.S. a little concerned V.S. very concerned)	CFQ<br>1) Restriction<br>2) Pressure to eat	1) +, *P* = 0.009<br>2) *P* = 0.517	Regression analyses<br>Child sex, age, race, parent weight and education status, household income status, and receipt of public assistance
Mallan, 2014 [76]	CFQ (three items)<br>Concern about child weight (Continuous variable)	CFQ<br>1) Pressure to eat<br>2) Restriction	1) β = 0.18 (SE: 0.05), *P* < 0.01<br>2) β = 0.10 (SE: 0.03), *P* < 0.01	Multiple linear regression<br>Child gender, age, and fathers' age, BMI, education.
Chae, 2018 [88]	One item<br>Concerns about children overweight or obese (concerned V.S. unconcerned)	Four items from the recommendations and the most frequently used items<br><br>Restriction	Adjusted OR = 2.89 (95% CI: 1.10, 7.615), *P* = 0.032	Multi-variate logistic regression analysis<br>Child weight status and maternal weight status, educational level, perception, and satisfaction with child weight.
Costa, 2021[23]	CFQ (one item)<br>Concern about child overweight (unconcerned V.S. concerned)	CFQ<br>1) Pressure to eat<br>2) Restriction	Unconcerned V.S. concerned at 4 y<br>1) β=-0.025 (95% CI: -0.130, 0.080)<br>2) β=0.226 (95% CI: 0.142, 0.310)<br>Unconcerned V.S. concerned at 7 y<br>1) β=0.050 (95% CI: -0.066, 0.166)<br>2) β=0.261 (95% CI: 0.169, 0.353)	Multiple linear regression<br><br>Covariates: maternal perception and dissatisfaction about weight, maternal education, child sex and zBMI
May, 2007 [71]	CFQ (one item)<br>Concern about child overweight (unconcerned V.S. concerned)	CFQ<br>1) Restriction<br>2) Pressure to eat	1) OR=5.94 (95CI: 1.74, 20.28)<br>2) Pressure to eat all: Adjusted OR=0.89 (95CI: 0.63, 1.26).<br>Pressure to eat enough: Adjusted OR=0.39 (95CI: 0.15, 0.99).<br>Pressure to eat right food: NR	Multiple logistic regression<br><br>Covariates: child sex, age and race/ethnicity

**First author, year**	**Exposure and measure**	**Outcome and measure**	**Result and Estimates**	**Statistical Analysis and covariates in the model**
Seburg, 2014 [72]	CFQ (three items)<br>Concern about child weight (Continuous variable)	CFQ<br>1) Restriction<br>2) Pressure to eat	1) b = 0.34, $P < 0.001$<br>2) NR	Multiple linear regression<br>Parent BMI and child age, sex, and BMI
Crouch, 2007 [78]	CFQ (three items)<br>Concern about child weight (Continuous variable)	CFQ<br>1) Restriction	1) β = 0.2, $P < 0.05$.	SEM<br>Covariates: Mothers' perceived responsibility, perception, education, BMI, perceived maternal weight; child gender and BMI; family income
Ayine, 2020 [73]	CFQ (three items)<br>Parental concern about child weight (Continuous variable)	CFQ<br>1) Restriction<br>2) Pressure to eat	1) r = 0.345, $P < 0.01$.<br>2) r = 0.08, $P > 0.05$.	Pearson's correlations<br><br>Not control for covariates
Jani Mehta, 2014 [25]	The NOURISH Questionnaire (one item)<br>Concerns about child weight (Concerned V.S. Not concerned)	CFPQ 1) Pressure to eat<br>CFQ 2) Restriction	1) Mean difference = -0.2 (95%: -0.4, 0.09)<br>2) Mean difference = -0.09 (95%: -0.3, 0.1)	Bivariate analyses<br><br>Not control for covariates
Nowicka, 2014 [82]	CFQ (three items)<br>Concern about child weight (Continuous variable)	CFQ<br>1) Restriction	1) β = 0.40, $P < 0.05$	SEM<br><br>Covariates: level of education, foreign background, and mother's and father's BMI

Notes. C-CFQ: Chinese Child Feeding Questionnaire; CFQ: Child Feeding Questionnaire; CFPQ: Comprehensive Feeding Practices Questionnaire; PFSQ: The Parental Feeding Style Questionnaire; PFQ: Preschooler Feeding Questionnaire; BMI: body mass index; SEM: Structural equation modelling; NR: not report; CI: confidence interval; SE: standard error.
